# Establishment and application of suspension static method in blood group screening of automated blood group analyzer

**DOI:** 10.1038/s41598-023-34495-z

**Published:** 2023-05-09

**Authors:** Min Huang, Chengping Ma, Yan Li, Ruiping Dong, Rongrong Pang, Shuizhen Huang, Qiang Fu, Libo Zhang

**Affiliations:** Department of Blood Screening Laboratory, Nanjing Red Cross Blood Center, Nanjing, Jiangsu China

**Keywords:** Coagulation system, Haematological diseases

## Abstract

The accuracy of blood group identification is the basis of blood transfusion safety. In order to increase the detection rate of weak agglutination, unexpected antibodies (UAb) and blood subtypes for pre-transfusion testing, the blood group screening process of automated blood group analyzer (ABGA) is ameliorated by introducing one static step and establishing a suspension static method (SSM). One static step was introduced in the blood group screening process of ABGA and three static time conditions were designed: 300 s, 400 s and 500 s, from which the optimal static time was selected and SSM was established; By comparing the detection of weak agglutination and UAb before and after the application of SSM, the feasibility and effect of suspension static method were verified and evaluated. The last two steps of the automatic blood group screening process were replaced with static, light centrifugation and imaging. The optimal static time parameter was selected as 400 s and SSM was established; After the application of SSM, it was verified that: (1) The detection level of weak antibodies (anti-A and anti-B) and weak antigens (weak D phenotype) could be improved by SSM, including antibodies in plasma of known type O samples with 0, 2, 4, 8, 16 and 32 times serial dilutions(simulating weak anti-A and weak anti-B), weak antibodies (anti-B) in plasma of one normal A-type sample and weak antigens on red blood cells (RBC) of 5 weak D phenotype samples (weak D antigen); (2) Three blood donor samples (type A, O and B) with known UAb were detected by SSM. The results showed that SSM could detect both weak antibodies (anti-A and anti-B) and UAb; (3) SSM was applied to detect the samples of 3 A_2_B and 3 subtype B blood donors and the blood subtypes could be clearly detected; (4) The number of screening samples was 95,314 and 106,814 before SSM (2018) and after (2020) the application of SSM and the positive rate of UAb (63/95,314 and 187/106,814) increased after SSM, discrepancy of which was statistically significant (*χ*^2^ = 48.42, *P* < 0.01). The above results demonstrate that SSM of ABGA is conducive to the detection of weak agglutination, UAb and blood subtypes in blood samples, which can improve the sensitivity of blood group detection and ensure the safety of clinical blood transfusion to a certain extent.

## Introduction

Despite the rarity of the clinical situations such as RBC donors carried UAb, when incompatible blood transfusion results in hemolytic transfusion reactions (HTRs), the consequences can be devastating. In the safety of blood transfusion, although the importance of blood type for blood donors and patients (recipients) should be viewed equally, patients are more likely to produce UAb, weak RBC antigen, etc.^1^. Hence the blood type of patients tends to be paid more attention. However, the blood type screening for blood donors is large in scale and most of them are healthy people. Meanwhile, the country does not have mandatory and clear regulations on UAb items in blood type screening. Thus the special blood type of blood donors is more likely to be missed in blood type screening, resulting in severe clinical events such as HTRs^2^. A large number of studies have shown that the screening of UAb before blood transfusion is very important and necessary for blood transfusion safety^3,4^. Therefore, in blood transfusion safety, the donor's blood group is the more important, in the prevention of allo immunisation and possible transfusion reactions (HTRs). Therefore, Blood group identification plays an extremely important role in blood testing of blood donors^5,6^. At present, ABGA is widely employed in blood banks for detection. The ABGA realizes complete automation and standardization from bar code scanning, sample transferring and dispensing, reagent dispensing to result interpretation, which reduces the influence of human factors on semi-automatic analyzers and is suitable for blood group detection of large quantity of samples in blood stations^7,8^. In addition to improvement of efficiency, documentation, traceability and archiving of results, the most prominent advantage of ABGA is recognized as the accuracy of blood group identification. Nevertheless, when blood samples containing weak antigen/antibody, UAb or subtype blood are screened, the ABGA may misjudge or fail to detect in blood group typing^9^.

The microplate technology uses automated platforms to detect serum antibodies and erythrocytes surface antigens. The reactants are centrifuged and incubated in microplates and the ABO/RhD blood type is read through an automated system. The blood group screening technology matched with ABGA is currently mainly micro-column gel method (MGM) and microplate technology. The microplate technology is widely used in various blood banks for its rapid, sensitive and low cost advantages^10^. Studies show that the accuracy of blood typing using U-shaped plates is as high as 99.6%, which is comparable to that of the test tube method^10^. At present, U-shaped plate method is used in our laboratory and the antigen–antibody ratio of blood typing procedure has already adjusted to the best through orthogonal test and manufacturer's recommendation. Compared with the results before adjustment, the present imaging results are clearer and the accuracy of blood group determination has been significantly improved (unpublished). However, in the actual work, we found that there are still deficiencies: because ABGA adopts the U-shaped plate method, after the conventional step of centrifugation and antigen–antibody interaction, it needs strong vibration to resuspend the agglutination block gathered in the center of the plate hole, the strong agglutination is still clustered in form of blocks or pieces, while the weak agglutination and non-agglutination will disperse and evenly suspend in the U-shaped plate hole, which is not easy to distinguish. The difficult point of U-plate method of ABGA is how to distinguish weak agglutination from non-agglutination and how to adjust the reaction process to make weak agglutination gather at the bottom of the hole, but non-agglutination is finally evenly dispersed in the hole. Therefore, this paper mainly discusses the establishment of U-shape microplate technology SSM and how to strengthen the detection ability of weak agglutination (including UAb and subtypes) in the process of blood group screening.

## Methods

### Ethics statement

This study conducted in accordance with the ethical standards set down by the declaration of Helsinki with its recent modification of 2013 (Fortaleza)^11^ was approved by the Ethics Committee of Nanjing Red Cross Blood Center (NJRCBCEC-2019–10). All participants have filled in the form of blood donor health consultation and signed the informed consent form of blood donors in writing before participating. The informed consent form of blood donors clearly states: "I agree to store the remaining blood samples tested in the laboratory and health-related information into the biological sample bank of healthy people in Nanjing Red Cross Blood Center, which can be used for human medical undertakings in the future." Therefore, the Ethics Committee of Nanjing Red Cross Blood Center allowed this study to be free of informed consent. The data involved in this study were only anonymous screening results and did not mention any information related to the privacy and identity of blood donors. The blood screening laboratory of this study has passed the review of China National Accreditation Commission Service for Conformity Assessment (CNAS) for medical laboratory in accordance with ISO15189. All staff of the laboratory has signed the confidentiality statement on the privacy and results of blood donors, which required that the privacy and identity information of blood donors be kept confidential.

### Sample sources

Sample sources of routine blood type screening work: vacuum blood collection tubes containing anticoagulant were used to collect 4–5 ml of venous blood of each unpaid blood donors in the center and centrifuged at 3500 rpm for 15 min.

Samples sources with known blood type: samples screened by ABGA as suspicious had been sent for confirming in the department of blood group laboratory of the blood center, including rare blood type samples: samples containing weak antigens and antibodies or UAb, samples of blood subtype such as 3 known blood subtype A_2_B and 3 known blood subtype B.

### Routine work process of ABGA

The sample plasma and 2–3% of the saline suspension containing sample RBC were respectively automatically and quantitatively dispense to the bottom of the 12 × 8-well U-shaped plate by the automated blood analyzer (STAR-BG; Hamilton, Switzerland), then the RBC reagents (ABO Reverse Typing Cassette; Shanghai Blood Biomedicine, Inc., Shanghai, China) were added to the sample plasma on plate, anti-A, anti-B(ABO Forward Typing Cassette; Shanghai Blood Biomedicine, Inc., Shanghai, China) and anti-D reagents(RhD IgM monoclonal reagent; Millipore, Livingston, West Lothian, UK) were added to the sample RBC on plate and 5 steps followed to finally determine the blood group results: mixing, centrifugation and antigen–antibody reaction, breaking up and re suspension, promoting sedimentation of agglutinated blocks, imaging. When agglutination occurs among the RBC, the agglutinated block settles and gathers in the center of the bottom of wells in the U-shaped plate, showing a bright red "cell button shape"; When agglutinate does not occur, the RBC are evenly dispersed and suspended in the whole well, showing a uniform pink " cloud and mist shape". The discrepant appearances of agglutination and non-agglutination are captured by charge-coupled device (CCD) of ABGA and converted into digital images, thus the analysis software judges the agglutination status of each well according to the imaging. The computer integrates the forward and reverse grouping of ABO blood group system and RhD imaging results to report the final blood group. If the forward and reverse typing results are consistent, then it will be determined as A, B, O and AB; If not consistent, then it will be determined as suspicious blood type and the samples will be delivered to department of blood group confirming laboratory to be further confirmed by standard tube technique and micro-column gel method (MGM). After conducting a series of these testing, if the blood type is suspected as subtype, further gene analysis shall be carried out.

### Establishing of SSM

The process of blood group screening by ABGA mainly includes 5 steps: mixing, centrifugation and antigen–antibody reaction, breaking up and resuspending, promoting sedimentation of agglutinated blocks and imaging. According to the recommendations of instrument and reagent manufacturers, our laboratory has determined the centrifugal speed and centrifugal duration parameters of each step: (1) mixing: 1200 rpm, 120 s; (2) centrifugation and antigen–antibody reaction: 550 rpm, 1200 s; (3) breaking up and resuspending: 1200 rpm, 45 s; (4) promoting sedimentation of agglutinated blocks: 350 rpm, 200 s; (5) Imaging. The manufacturers allow various laboratories to adjust parameters within a certain range according to specific conditions. “Mixing” adopts the pattern of strong agitation characterized by short-time and high-speed, which aims to fully mix the sample and reagent and make sure they are in full contact; “centrifugation and antigen–antibody reaction” adopts a mild pattern of long-term and low-speed, which aims to fully react the blood group antigens on the cell surface with the antibodies and form agglutinated blocks; The pattern of strong agitation as short-time and high-speed adopted to “breaking up and resuspending”, shatters and scatters the agglutinated blocks and resuspends the cells that do not agglomerate but have settled, so as to prepare for the further distinction between agglutination and non-agglutination; Before imaging, the agglutinated blocks gradually sedimentate to the bottom of the well in U-shaped plate at a mild speed and the non-agglutination is still evenly dispersed in the well showing a "cloud and mist shape". In this step, although strong agglutination and non-agglutination can be distinguished, weak agglutination and non-agglutination are still dispersed in the well in a "fine sand shape", which is difficult to distinguish. If we reduce interference caused by physical vibration to the weak agglutination before imaging, make it in a completely static state and prolong the settlement time of weak agglutination, is it possible to make the weak agglutination settle more at the well bottom and distinguish it from non-agglutination? Therefore, 3 settlement durations were designed in this study: 300 s, 400 s and 500 s. By observing the imaging results caused by discrepant settlement durations, the time easiest to distinguish between weak agglutination and non-agglutination was selected as the best static time. After this standing step, light centrifugation at 350 rpm for 30 s (this parameter comes from the recommendation of Hamilton manufacturer. This centrifugation condition can accelerate the aggregation of weak agglutination and resuspend the settled non-agglutinated cells) could be used. Therefore, the established complete SSM was divided into five steps: (1) mixing, (2) centrifugation and antigen–antibody reaction, (3) breaking up and resuspending, (4) standing, (5) light centrifugation and imaging. That is, the original step of “promoting sedimentation of agglutinated blocks” was replaced with “standing” and then “light centrifugation and imaging” were added. In order to observe the agglutination status of various antigens and antibodies as much as possible, 3 blood donors’ samples were selected including 2 normal blood type samples (AB-type and O-type) and one UAb-positive AB-type sample. ABGA parameters were adjusted before blood type detecting, static step was added with the static time parameter set to 300 s, 400 s and 500 s respectively. After light centrifugation step (350 rpm and 30 s), imaging was performed.

### Validation of SSM

#### Detection of weak antibodies (anti-A and anti-B) and weak antigens (anti-D) by SSM

Normal AB-type plasma was added for dilution into known O-type plasma which containing normal antibodies (anti-A and anti-B). O-type plasma of blood samples was diluted in series by 0, 2, 4, 8, 16 and 32 folds, then the simulated weak antibodies(anti-A and anti-B) were mixed with ABO RBC reagents which contained A1 cells, B cells, O cells. Then the established SSM was applied to the detection of the simulated weak antibodies in reverse typing, aiming to verify SSM. The maximum dilution ratio of blood type antibody that could be just detected by ABGA was observed in the imaging results. Routine work process and SSM were respectively applied to the detection of one normal A-type sample (contrast) and one A-type sample with weak anti-B which had been confirmed and then the imaging results by routine work process and SSM were observed and compared. Routine work process and SSM were respectively applied to the detection of weak antigens (RhD) of 5 known weak D phenotype donors’ samples in forward typing, including 2 cases of A-type, one case of B-type, one case of O-type and one case of AB-type. The imaging results by routine work process and SSM were observed and compared.

#### Detection of UAb by SSM

Routine work process and SSM were respectively applied to forward and reverse typing of 3 donors’ samples with A_2_-subtype, O-type, B-subtype, , all of which had been confirmed as containing UAb. The imaging results by routine work process and SSM were observed and compared.

#### Detection of blood subtypes by SSM

Routine work process and SSM were respectively applied to the forward and reverse typing of 6 blood donors’ samples with confirmed blood subtype: 3 were A_2_B-subtype and 3 were B-subtype. The imaging results by routine work process and SSM were observed and compared.

### Comparison of results via high-throughput blood group screening before (2018) and after (2020) application of SSM

In this study, 95,314 samples of voluntary blood donors were screened in 2018 (after the application of routine work process and before the application of SSM) and 106,814 in 2020 (after the application of SSM) by ABGA. The samples with suspicious results were sent to the department of blood type laboratory for further confirming and finally confirmed by standard tube technique (such as absorption and release test), MGM, gene sequencing and other classical methods. Some of the suspicious samples were confirmed to be positive for UAb. The total calculated number of UAb positive included the number of UAb positive outside the ABO blood group system and the number of ABO blood subtype antibody positive. Submission rate = (number of samples submitted for confirming/number of samples screened) × 100%, submission conformity rate = (number of UAb-positive samples/number of samples submitted for confirming) × 100%, UAb-positive rate = (number of UAb-positive samples/number of samples screened) × 100%.

### Statistical analysis

The percentage of discrepant results of ABGA was calculated. The results of blood screening were automatically derived by laboratory software. The comparison between discrepant rates was statistically analyzed with chi square test by SPSS software (SPSS Statistics, ver. 25; IBM, Armonk, NY, USA). *P* value < 0.01 was considered statistically significant.

## Results

### The preliminary establishment of SSM

**Figure 1:**
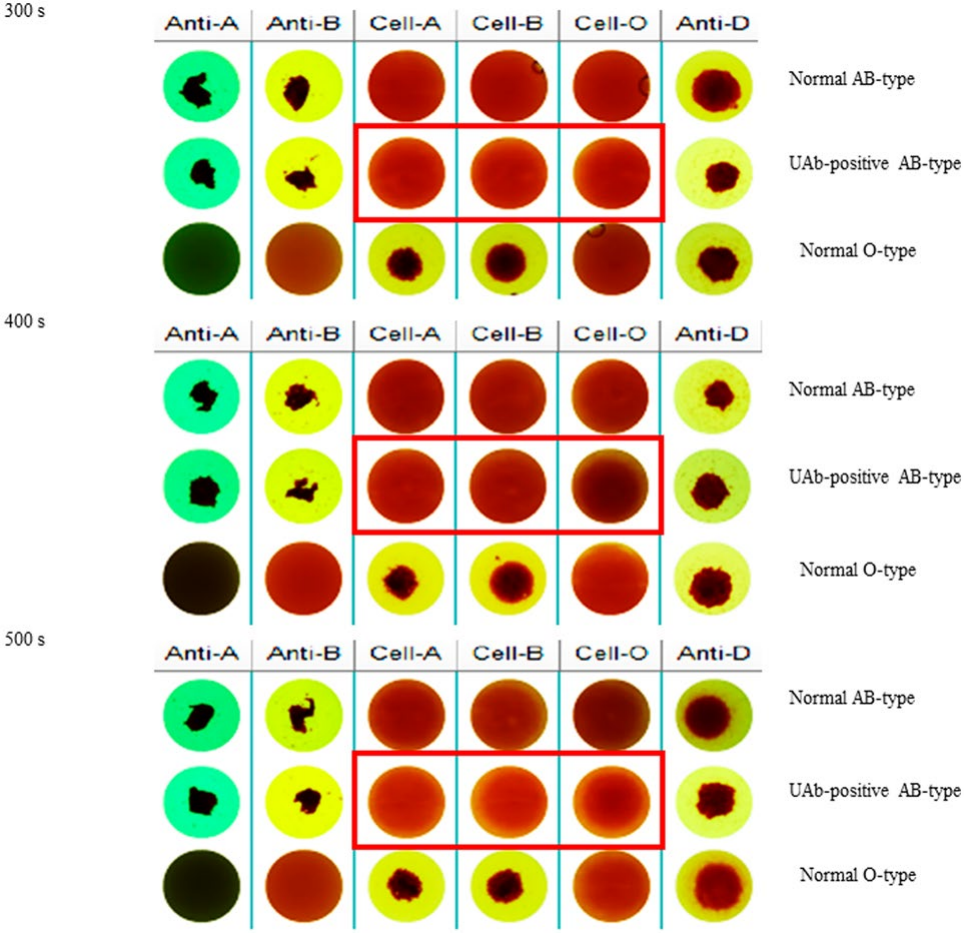
Imaging results of ABGA with 3 discrepant parameters. 300 s, Imaging results after standing 300 s by SSM; 400 s, Imaging results after standing 400 s by SSM; 500 s, Imaging results after standing 500 s by SSM. Normal AB-type, known normal AB-type donor’s sample; UAb-positive AB-type, known UAb-positive AB-type donor’s sample; Normal O-type, known normal O-type donor’s sample. *Anti-A* anti-A reagent, *Anti-B* anti-B reagent, *Cell-A* A1 phenotype red cell reagent, *Cell-B* B phenotype red cell reagent, *Cell-O* O phenotype red cell reagent, *Anti-D* anti-D reagent.

The final imaging results of ABGA were shown in Fig. 1. The reverse typing imaging results with regard to UAb-positive AB-type as shown in the red square frame displayed that, neither non-agglutination nor weak agglutination subsided after 300 s; after 400 s, there was still no sedimentation with non-agglutination (column "Cell-A" and "Cell-B"), but the weak agglutination (column "Cell-O") had begun to subside distinctly; after 500 s, both weak agglutination and non-agglutination had sedimentation. Therefore, the static time parameter of SSM was selected as 400 s.

### Weak antibodies and antigens could be detected better by SSM

**Figure 2:**
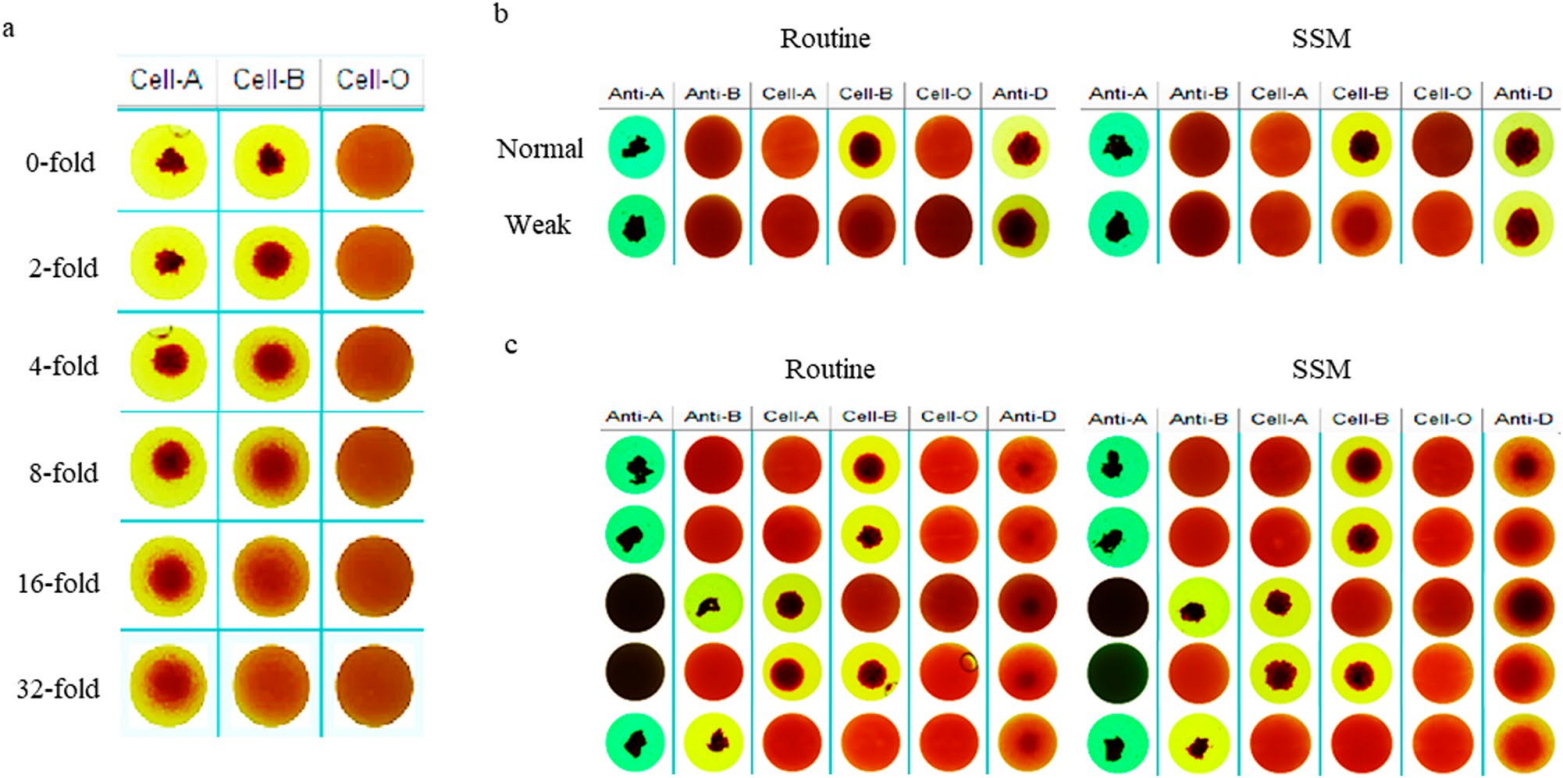
Detection results of weak antibodies and antigens by SSM. (**a**) Detection of simulated antibodies (anti-A and anti-B) by SSM. (**b**) Detection of real weak antibodies (anti-B) by SSM. (**c**) Detection of weak antigens (anti-D) by SSM. 0-fold, known O-type plasma without diluting; twofold, known O-type plasma of twofold diluted with AB-type plasma; fourfold, known O-type plasma of fourfold diluted with AB-type plasma; eightfold, known O-type plasma of eightfold diluted with AB-type plasma; 16-fold, known O-type plasma of 16-fold diluted with AB-type plasma; 32-fold, known O-type plasma of 32-fold diluted with AB-type plasma. *Routine* Routine work process, *SSM* suspension static method, *Normal* Known normal A-type donor’s sample, *Weak* Known A-type donor’s sample with weak anti-B, *Anti-A* anti-A reagent, *Anti-B* anti-B reagent, *Cell-A* A1 phenotype red cell reagent, *Cell-B* B phenotype red cell reagent, *Cell-O* O phenotype red cell reagent, *Anti-D* anti-D reagent.

Weak anti-A in plasma was well detectable even after 32-fold dilution, while anti-B could be detected after eightfold dilution but became undetectable after 16-fold dilution (Fig. 2a); The results of A-type normal plasma containing weak antibodies (anti-B) were shown in Fig. 2b. Compared with the results of routine work process, the weak agglutination image of anti-B (column "Cell-B") in SSM results is clearer (Fig. 2b); The SSM imaging results of 5 cases of weak D phenotype illustrated more agglutination and sedimentation of antigens with weak D phenotype (column "Anti-D") and appeared clearer compared with the results of conventional work process (Fig. 2c).

### UAb could be detected better by SSM

**Figure 3:**
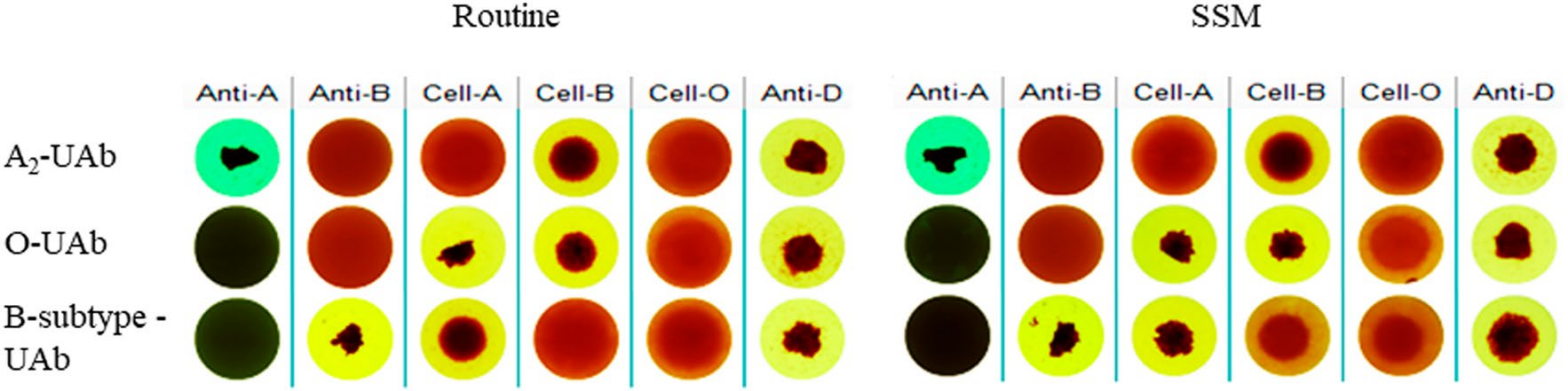
Detection results of UAb by SSM. *Routine* Routine work process, *SSM* suspension static method, *A*_*2*_*-UAb* Known A_2_-subtype donor’s sample with UAb, *O-UAb* Known O-type donor’s sample with UAb, *B-subtype-UAb* Known B-subtype donor’s sample with UAb, *Anti-A* anti-A reagent, *Anti-B* anti-B reagent, *Cell-A* A1 phenotype red cell reagent, *Cell-B* B phenotype red cell reagent, *Cell-O* O phenotype red cell reagent, *Anti-D* anti-D reagent.

It could be seen from Fig. 3 that SSM could detect not only weak anti-A and anti-B but also UAb (column "Cell-O"). Compared with the results of routine work process, the weak agglutination image of UAb (column "Cell-O") in SSM results has stronger agglutination intensity and the image is clearer and easier to judge (Fig. 3).

### SSM could better detect blood subtypes

**Figure 4:**
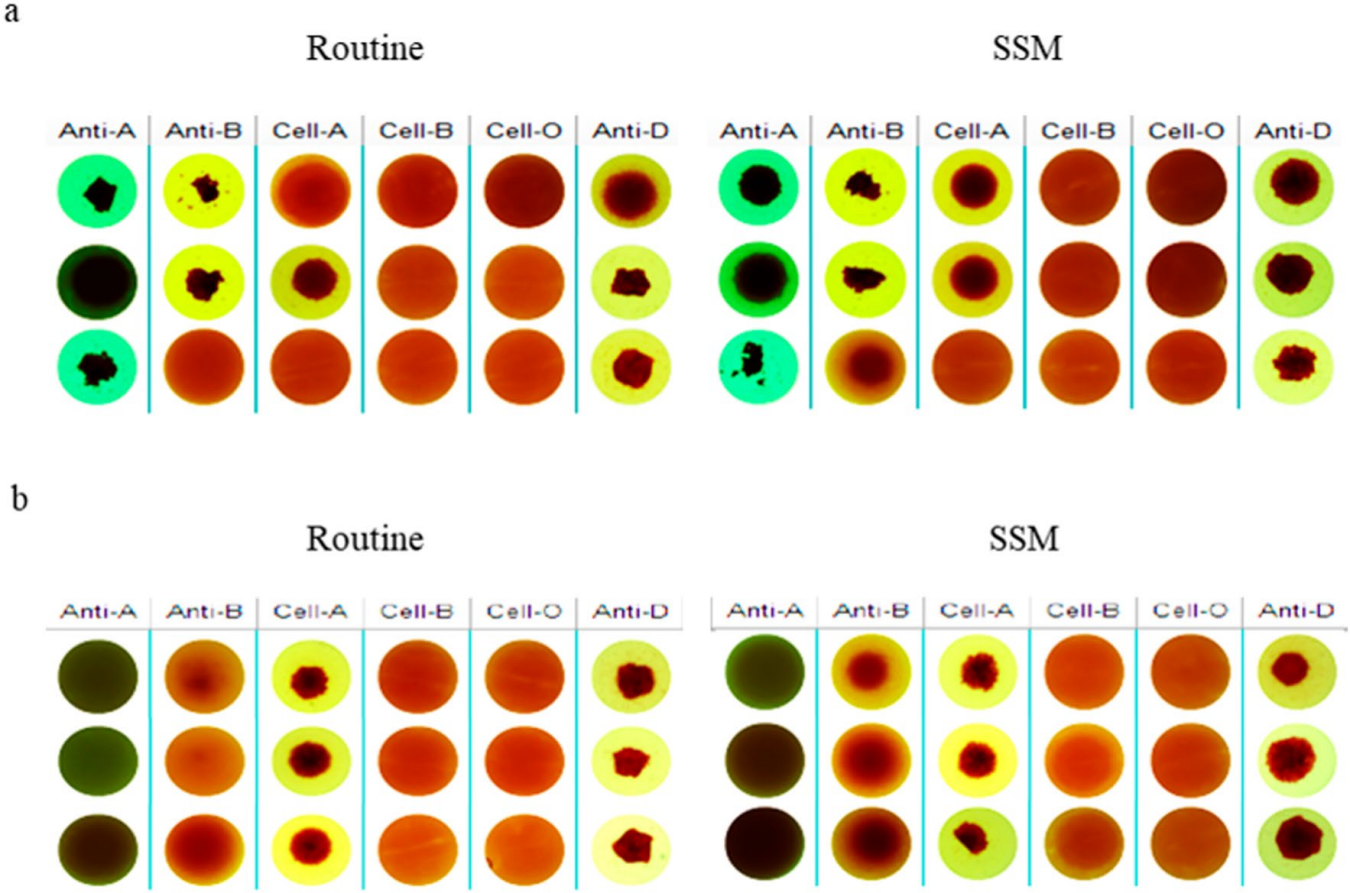
Detection results of blood subtypes by SSM. (**a**) 3 known A_2_B-subtype donors’ samples. (**b**) 3 known B-subtype donors’ samples. *Routine* Routine work process, *SSM* suspension static method, *Anti-A* anti-A reagent, *Anti-B* anti-B reagent, *Cell-A* A1 phenotype red cell reagent, *Cell-B* B phenotype red cell reagent, *Cell-O* O phenotype red cell reagent, *Anti-D* anti-D reagent.

It could be seen from Fig. 4 that, the weak agglutination phenomenon in blood subtypes could be more distinctly observed in SSM results compared with routine work process results, including anti-A1 (column "Cell-A") and weak B antigen (column "Anti-B") in A_2_B-subtypes (Fig. 4a) and also including weak B antigen (column "Anti-B") and weak B antibody (column "Cell-B") in B subtypes (Fig. 4b).

### Comparison of results via high-throughput blood group screening before (2018) and after (2020) application of SSM

The number of samples screened was 95,314 and 106,814 before the application of SSM (2018) and after the application of SSM (2020) (Table 1). The number of samples submitted for confirming was 235 (0.25%) and 850 (0.80%) respectively, suggesting that the proportion of suspicious samples found in the preliminary screening of ABGA increased after the application of SSM, which was distinctly statistically significant (*P* < 0.01). The number of UAb-positive samples were 63 and 187 respectively with no significant difference in the submission conformity rate (63/235 and 187/850), suggesting that the true positive rate of suspicious samples found by ABGA was stable at a certain level and did not increase. The UAb-positive rate (63/95,314 and 187/106,814) increased after the application of SSM, which was distinctly statistically significant, suggesting that the overall true positive rate of the preliminary screening results of ABGA increased after the application of SSM, which might be caused by the increase of the submission rate.

**Table 1:** Comparison of blood group screening before (2018) and after (2020) the application of SSM.

Year	Number of samples screened		Number of samples submitted for confirming		Rate of samples submitted for confirming		Number of UAb-positive samples		conformity rate of samples submitted for confirming			Rate of UAb-positive samples
2018		95,314		235		0.25%		63		26.81%	0.07%	
2020		106,814		850		0.80%		187		22.00%	0.18%	
*χ*^2^						284.57				2.4	48.42	
*P*						< 0.01				0.12	< 0.01	

## Discussion

In a broad sense, "UAb" refer to the antibodies other than the antibodies of ABO blood group system, including the antibodies of ABO subtype and the antibodies of non ABO blood group system. People can obtain UAb by infusing non-self blood products or during pregnancy due to the presence of fetuses with discrepant blood groups. In a few cases, there will be a lack of obvious stimulating factors. At present, the total positive rate of UAb of blood donors in China is 0.20%^12,13^, which is close to that of this study (0.18%). It was reported that the positive rate of UAb screened by MGM in Harbin was 0.0049%^14^, which was significantly lower than that of U-plate method. Generally, the sensitivity of the two methods should be similar and this considerable difference may be caused by regional differences or other confounding variables. According to reports in Hong Kong, two different systems were used to screen blood donors for UAb, with detection rates of 0.1% and 0.55%. The former (PK7300) was slightly lower than the results of this study (0.18%), while the latter (IH-1000 System) was significantly higher than the results of this study^14^. This indicates that there may be discrepancies in results between different regions and different systems with various analyzers and methods. RH blood group system accounts for the largest proportion of UAb^15^. The significance of RhD blood group in clinical blood transfusion is second only to ABO blood group. Severe transfusion reactions may occur if the blood of RhD positive or weak D donors is transfused into RhD negative donors^16^. Although the detection rate of UAb in the population is not high, it is one of the main reasons for HTRss, hemolytic disease of the fetus and newborn (HDFN), difficulty in blood group identification and in blood cross matching^17^. As a routine test before blood transfusion, UAb screening has been carried out abroad many years ago and its results have also been reported^18^. Many domestic hospitals have also carried out UAb screening. For blood banks, blood group screening for UAb has become the first assurance for clinical blood transfusion safety.

Subtype A is the most common in ABO blood group system. Subtype A mainly includes A_1_ (80%) and A_2_ (20%) accounting for 99.9% of all type A blood; the other subtypes A (A_3_, A_x_, A_m_) are few^19^. Subtype B (B_3_, B_m_, B_x_) is less common than subtype A and has little clinical significance^20^. About 0.4% of A_2_ and 25% of A_2_B samples contain anti-A_1_^21^. The results of this study illustrated that (Fig. 2a), anti-A in plasma could still be well detected after 32-fold dilution and anti-B could still be well detected after eightfold dilution. Figures 2b, 3 and 4b showed that weak anti-B could also be detected by SSM. It could be seen that SSM had a very excellent detection effect for common subtypes (Fig. 4a) and also has a good detection effect for rare subtype B.

Weak agglutination brings challenges to blood group screening in blood banks. At present there are no domestic laws and regulations explicitly requiring antibody screening for blood donors, most institutions use ABGA adopting saline method and standard “O-type erythrocytes” for screening, but there is no clear illustration for the blood group antigens contained on “O-type erythrocytes” surface. Generally, blood collection and supply institutions can only randomly screen some UAb of strong agglutination with saline method. The weaker agglutination produced by weak antigens and antibodies in blood group samples is easier to be missed by automatic equipment during screening, which increases the unreliability of blood group detection^9,22^. Interaction of antigen–antibody during blood group screening is not only affected by the ratio of antigen to antibody and self binding force, but also affected by the buoyancy of reaction medium. In the weak agglutination phenomenon, the blood group antigen on the surface of RBC can actually react with the antibody in plasma, but the intensity is weak and the small agglutination block formed is not easy to be observed and recognized directly. According to Stokes formula, the settling velocity of solid particles in liquid depends on particle size and liquid properties. The settlement of particles in vacuum is not affected by any resistance, but only by gravity and moves in free fall. However, the settlement of particles in medium is not only affected by gravity, but also affected by the viscous resistance of medium opposite to gravity^23^, that is, in addition to immunological agglutination reaction, gravity and buoyancy factors are also involved in the reaction system.

Therefore, increasing the proportion of plasma antibody in the interaction system of antigen–antibody can theoretically increase the agglutination of weak antigen, but increasing the amount of plasma will increase the density and buoyancy of the reaction system, that is, the resistance of the medium will increase and the sedimentation speed will slow down, which will lead to the difficulty of sedimentation of weak agglutination, which is not conducive to the detection of weak agglutination. At the same time, due to the increase of plasma volume, the imaging results will also be disturbed for samples with lipid or hemolysis blood samples. Moreover, before this study, the laboratory has already adjusted the proportion of antigen and antibody to the best. Therefore, it is impossible to further increase the detection of weak agglutination simply by increasing the proportion of plasma volume.

Since the adjustment of antigen–antibody ratio can not improve the detection rate of weak agglutination, can adjusting the centrifugal parameters involved in the reaction step improve the detection rate of weak agglutination? Studies have shown that the sensitivity of screening can indeed be increased by adjusting the parameters of ABGA^24^. ABGA generally adopts the steps of mixing, centrifugation and reaction, breaking up and resuspension, promoting agglutination blocks sedimentation and imaging for blood group detection. Each step is set with a certain centrifugal force and duration. As long as a certain centrifugal force and reaction time are reached, the blood group antigen–antibody can carry out sufficient binding reaction, so the speed and time of centrifugation are not the key parameters affecting the test. This study found that the key step is how to treat after disperse and resuspension: make the weak agglutination completely stand and prolong its sedimentation time, which has a significant effect on improving the detection rate of weak agglutination. The application of ABGA has been very common all over the world^9,25^. On account of ABGA system is an open and flexible platform, the staff in various regions can cooperate with the instrument manufacturers and increase the sensitivity of blood group weak agglutination detection by prudently adjusting the screening process. Therefore, the results of this study also provide a certain reference value for blood group screening in other regions.

Regarding Table 1, there were a few potential confounding variables influencing submission rates and UAb-positive rate between 2018 and 2020. The most likely confounding variable is that the increase in the number of samples submitted for confirming leads to an increase in the number of UAb-positive samples. However, from the results in Table 1, it can be seen that the submission conformity rate has not increased, but has mildly decreased. It can be seen that the number of samples submitted for confirming and the number of UAb -positive samples have not increased in proportion. The second possible confounding variable is that the increase in the number of samples screened causes an increase in the number of UAb -positive samples, but using expression with ratio (0.07% vs 0.18%) can avoid this problem. Although the number of samples screened and the number of UAb -positive samples are rising in sync, the number of UAb -positive samples is rising more. The third potential confounding variable is the increase in the number of COVID-19 infections in 2020. Whether viruses, drugs, or vaccines can cause an increase in the number of UAb-positive cases in the population is unknown, but blood donors usually undergo a rigorous process of health consultation and the impact of the virus will be minimized to the greatest extent.

Finally, it was worth noting that from the results in Table 1, it could be seen that the coincidence rate of submission for confirming had not been improved after the application of SSM. Although the sensitivity of blood group weak agglutination detection could be increased by adjusting the screening process, but the specificity still needs to be further improved by means of antibody screening test, suggesting that the blood group screening of blood donors by blood stations was only the first step in the safety of clinical blood transfusion. The challenges of weak agglutination and UAb to the safety of clinical blood transfusion need the joint cooperation and efforts of both the blood stations and the hospitals.

## Data Availability

Data are available upon reasonable request per institutional policy and Min Huang (Email:huangmmin@sina.com) should be contacted if someone wants to request the data from this study.

## Abbreviations

UAb: Unexpected antibodies
ABGA: Automated blood group analyzer
SSM: Suspension static method
MGM: Micro-column gel method
HTRs: Hemolytic transfusion reactions
HDFN: Hemolytic disease of the fetus and newborn
CCD: Charge-coupled device
RBC: Red blood cell
